# Peritoneal dissemination of prostate cancer due to laparoscopic radical prostatectomy: a case report

**DOI:** 10.1186/1752-1947-5-355

**Published:** 2011-08-05

**Authors:** Yoshiki Hiyama, Hiroshi Kitamura, Satoshi Takahashi, Naoya Masumori, Tetsuya Shindo, Mitsuhiro Tsujiwaki, Tomoko Mitsuhashi, Tadashi Hasegawa, Taiji Tsukamoto

**Affiliations:** 1 Department of Urology, Sapporo Medical University School of Medicine, Sapporo, Japan; 2 Department of Surgical Pathology, Sapporo Medical University Hospital, Sapporo, Japan

## Abstract

**Introduction:**

Peritoneal dissemination with no further metastases of prostate cancer is very rare, with only three cases reported in the available literature. We report the first case of iatrogenic peritoneal dissemination due to laparoscopic radical prostatectomy.

**Case Presentation:**

A 59-year-old Japanese man underwent laparoscopic radical prostatectomy for clinical T2bN0M0 prostate cancer, and the pathological diagnosis was pT3aN0 Gleason 3+4 adenocarcinoma with a negative surgical margin. Salvage radiation therapy was performed since his serum prostate-specific antigen remained at a measurable value. After the radiation, he underwent castration, followed by combined androgen blockade with estramustine phosphate and dexamethasone as each treatment was effective for only a few months to a year. Nine years after the laparoscopic radical prostatectomy, computed tomography revealed a peritoneal tumor, although no other organ metastasis had been identified until then. He died six months after the appearance of peritoneal metastasis. An autopsy demonstrated peritoneal dissemination of the prostate cancer without any other metastasis.

**Conclusion:**

Physicians should take into account metastasis to unexpected sites. Furthermore, we suggest that meticulous care be taken not to disseminate cancer cells to the peritoneum during laparoscopic radical prostatectomy.

## Introduction

Peritoneal dissemination with no further metastases of prostate cancer is very rare with, to the best of our knowledge, only three cases reported in the available literature. There has not yet been a report of a patient undergoing surgical treatment that might have resulted in iatrogenic dissemination. We report the first case of iatrogenic peritoneal dissemination due to laparoscopic radical prostatectomy (LRP).

## Case presentation

A 59-year-old Japanese man presented to our urology clinic with lower urinary tract symptoms. His serum prostate-specific antigen (PSA) level was 9.5 ng/mL. A digital rectal examination revealed a hard induration of his prostate. He had no personal or familial history of malignant disease. A prostate biopsy was performed and showed Gleason score 3+4 adenocarcinoma of the prostate. Computerized tomography (CT) and bone scintigraphy showed no metastasis. He was referred to our Department of Urology for treatment of cT2bN0M0 prostate cancer, and underwent LRP. The operation was performed with a transperitoneal approach. The pathological diagnosis was pT3aN0 Gleason score 4+4 adenocarcinoma with a positive surgical margin.

After the operation, his PSA level dropped to 0.7 ng/mL at its lowest, and so salvage radiation therapy with 50 Gy was carried out. His serum PSA level initially dropped to 0.5 ng/mL but began to increase, to 3.5 ng/mL, shortly after. Medical castration was then started. The therapy was effective for 24 months, after which he needed additional anti-androgen agents (bicalutamide and flutamide) and estramustine phosphate because of an increase in his PSA level. Sixty-six months after the prostatectomy (PSA 76.3 ng/mL) dexamethasone was administered, and provided the minimal PSA level, 0.58 ng/mL, 18 months after the initial administration. However, his PSA level increased again, so the endothelin receptor antagonist was replaced by dexamethasone for 12 months with no effect on his PSA level. Thereafter dexamethasone was administered again, and his PSA decreased from 340 ng/mL to 118 ng/mL.

**Figure 1:**
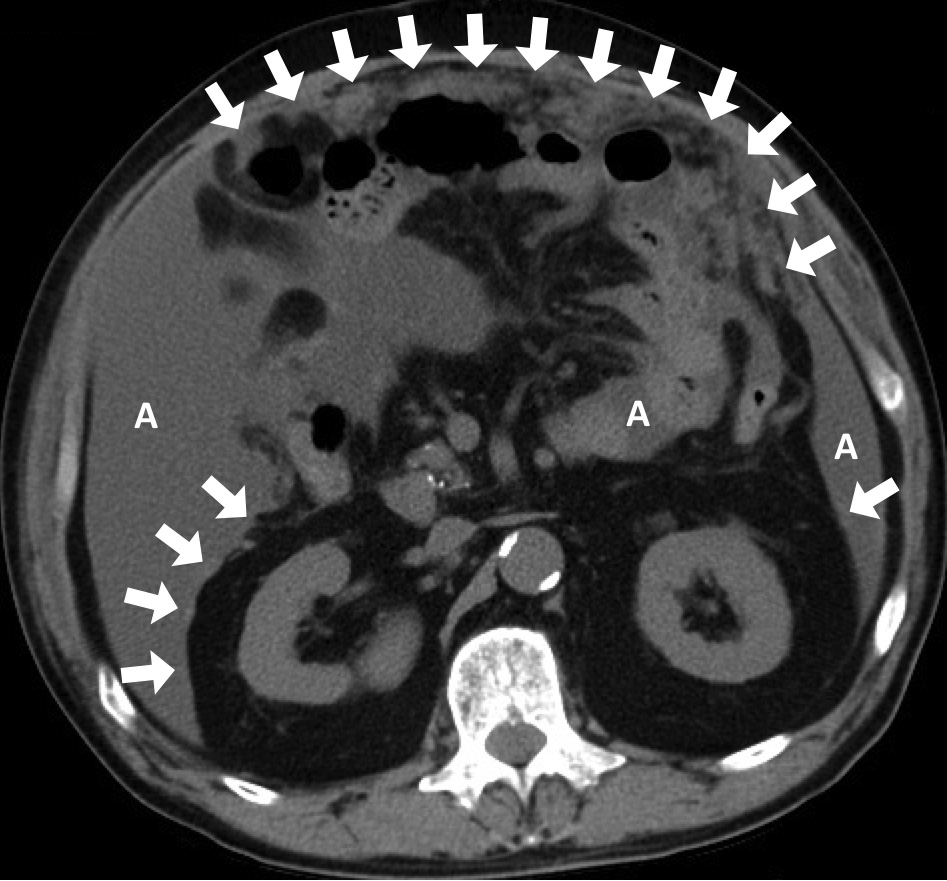
**An abdominal CT scan shows mesenteric metastases (arrows) and ascites (A) due to peritoneal dissemination**.

After that, his PSA level continued to increase without any metastasis visible on CT or bone scans. Our patient could not undergo chemotherapy with docetaxel because of complications with heart failure and interstitial pulmonary disease. At age 69, 114 months after the LRP, CT showed a peritoneal tumor that was considered to be a peritoneal metastasis (Figure 1). His PSA level was 168 ng/mL, and no other organ metastasis was found. Five months later, metastases to the mesentery were revealed by CT. The peritoneal metastases progressed with a large amount of ascites, and our patient died 120 months after the LRP.

**Figure 2:**
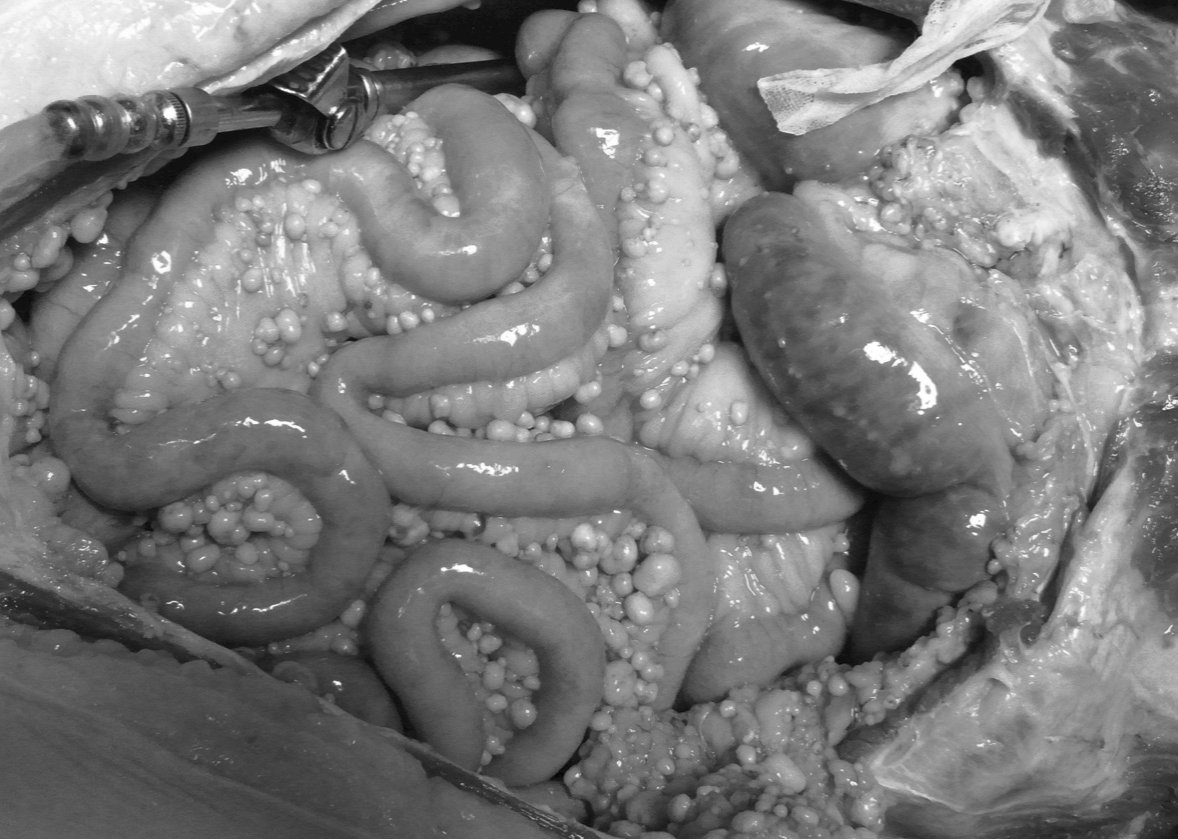
**Multiple nodules in the mesentery at autopsy**.

An autopsy revealed 4000 ml of clear yellow ascites and numerous nodules in his peritoneum, mesentery and omentum (Figure 2). These were pathologically diagnosed as dissemination of prostate cancer. No other metastasis was detected in any organ in the pathological evaluation. There was no port-site metastasis, during follow-up or at autopsy.

## Discussion

Metastases from prostate cancer to the bone, lymph nodes and lung are common events, but peritoneal metastasis is very rare and seldom reported in the literature. Even at autopsy, peritoneal metastasis is unusual, whereas bone (90%), lung (46%), liver (25%), pleural (21%) and adrenal (13%) metastases are reported in some large autopsy series [1]. Only three cases with peritoneal metastasis from prostate cancer have been reported (Table 1) [2-4]. Although these three cases had no opportunity for tumor implantation, our patient might have incurred iatrogenic dissemination to the peritoneum during the LRP. To our knowledge, this is the first case of iatrogenic peritoneal dissemination due to LRP. The main causes of such metastases appear to be tumor behavior and laparoscopy-related factors [5,6], including gas ambience [7], surgical manipulation [6] and overuse of ultrasonic scissors [8]. Alternatively, the dissemination may have been due to poor surgical technique, since this was only the second case of LRP in our institute. Lee *et al. *reported that poor technique increased port-site metastasis risks [9] and growing experience decreases this incidence [10]. However, the possible existence of peritoneal metastases at the LRP cannot be ruled out, since his serum PSA level did not fall under the lowest measuring limit during the local therapies.

**Table 1:** Summary of reported cases of peritoneal metastasis of prostate cancer

Authors	Age	Initial PSA (ng/mL)	Gleason score	Initial TNM	Treatment before detection of the peritoneal metastasis	PSA at the diagnosis of peritoneal metastasis (ng/mL)	Treatment after the diagnosis of peritoneal metastasis	Follow-up after the diagnosis of peritoneal metastasis
Kehinde *et al. *[2]	76	365	4+4, mucinous adenocarcinoma	T3(?)N0M1	-	365	Hormone therapy	18 months, AED

Brehmer *et al. *[3]	75	42	4+5	T3N0M1	-	42	Hormone therapy	14 months, AED

Zagouri *et al. *[4]	75	33	4+5	T×N0M0	Hormone therapy for 72 months	74	Docetaxel + estramustine phosphate	18 months, AED

Present case	69	9.5	4+4	T3aN0M0	Radical prostatectomy, salvage radiotherapy, and hormone therapy for 89 months	168	Palliative	6 months, DOD

AED, alive with evidence of disease; DOD, dead of disease

The pathological diagnoses of the previous three cases were Gleason 4 and/or 5 adenocarcinoma with or without mucinous adenocarcinoma (Table 1). Two of them demonstrated good responses to hormone therapy [2,3], and the combination of docetaxel with estramustine phosphate was effective in the other case [4]. Our patient experienced 120-month survival after the initial treatment, although no therapy was available without dexamethasone when the peritoneal metastasis was detected. Thus the standard strategy should be considered as a treatment for peritoneal metastasis from prostate cancer.

## Conclusion

Peritoneal dissemination of prostatic carcinoma is a very rare occurrence. Meticulous procedures during LRP should be performed to avoid a dissemination of cancer cells to the peritoneum. The treatment should be performed in accordance with the standard strategy for prostate cancer, including hormone therapy and chemotherapy.

## Consent

Written informed consent was obtained from the patient for publication of this case report and accompanying images. A copy of the written consent is available for review by the Editor-in-Chief of this journal.

## Abbreviations

CT: computerized tomography; LRP: laparoscopic radical prostatectomy; PSA: prostate-specific antigen.

## Competing interests

The authors declare that they have no competing interests.

## Authors' contributions

HY, HK, ST, NM, TS and TT were involved in conception, design and interpretation. HY and HK wrote the manuscript. MT, TM and TT performed the histological examination and provided the histopathological images. All authors read and approved the final version submitted.
